# Social Networks, Health Support, and Dietary Intake in Mothers Receiving Home Visiting Services

**DOI:** 10.1007/s40615-025-02286-z

**Published:** 2025-01-22

**Authors:** Sydney Miller, Sarah‑Jeanne Salvy, Nenette Caceres, Trevor Pickering, Wandi Bruine de Bruin, Tom W. Valente, John P. Wilson, Kayla de la Haye

**Affiliations:** 1 Keck School of Medicine, University of Southern California, Los Angeles, USA; 2 Dornsife School of Public Health, Drexel University, Philadelphia, PA 19104, USA; 3 Research Center for Health Equity, Samuel Oschin Comprehensive Cancer Institute, Cedars-Sinai Medical Center, Los Angeles, USA; 4 Center for Economic and Social Research, Arts and Sciences, Dornsife College of Letters, University of Southern California, Los Angeles, USA; 5 Sol Price School of Public Policy and Dornsife Department of Psychology, University of Southern California, Los Angeles, USA; 6 Schaeffer Center for Health Policy and Economics, University for Southern California, Los Angeles, USA; 7 Spatial Sciences Institute, Arts and Sciences, Dornsife College of Letters, University of Southern California, Los Angeles, USA; 8 Viterbi School of Engineering and the School of Architecture, University of Southern California, Los Angeles, USA; 9 Department of Psychology, Arts and Sciences, Dornsife College of Letters, University of Southern California, Los Angeles, USA

**Keywords:** Social networks, Health support, Health undermining, Diet disparities, Maternal health

## Abstract

Home visiting programs (HVPs) provide services to pregnant individuals and parents of young children to improve families’ health and well-being. However, little is known about these families’ social contexts. This study explores the social networks and dietary intake of mothers enrolled in a HVP, focusing on health support and health undermining. Cross-sectional data from 76 mothers enrolled in a HVP in Los Angeles County were collected by interview, using validated measures. Almost all mothers (95.7%) had one or more health supporters, while 55.1% had one or more health underminers. Some key findings related to health support were that mothers with higher income had more health supporters in their network (*b* = 1.36, *p* = 0.03), and network members were more likely to be health supporters if they were a romantic partner (OR = 3.41, *p* < 0.001), a resource-based connection (OR = 3.46, *p* < 0.01), or if they lived in the same neighborhood as the mother (OR = 1.68, *p* < 0.05). Further, having a health supporter who lived in the same neighborhood was associated with consuming more (1 + daily servings) vegetables (OR = 3.0, *p* < 0.05) and no sugar-sweetened beverages (OR = 0.29, *p* < 0.05). There were fewer findings related to health undermining: network members more likely to be underminers were romantic partners (OR = 8.93, *p* < .0001), and those perceived as having overweight or obesity (OR = 3.98, *p* < 0.001), but health undermining did not predict dietary intake. Overall, a broad set of network features were linked with health support, and given that some types of support were linked with better diet, network-based diet interventions leveraging health support may be effective in this priority population.

## Introduction

Home visiting programs (HVPs) provide support and services to hundreds of thousands of caregivers, families, and infants across the United States (U.S.) and are broadly focused on improving overall health and well-being [1, 2]. Over 50% of families receiving HVP services are living below the federal poverty level and cumulate sociostructural risks for poor maternal and child outcomes (e.g., housing instability, minoritized race and/or ethnicity, lack of childcare) [3]. Given the intersection of socio-structural barriers based on gender, race and ethnicity, and socioeconomic status [4–8], families enrolled in HVPs likely face barriers for healthy dietary intake. HVPs therefore represent an opportune avenue for interventions that foster healthy dietary behaviors, as they provide many social and health-based resources that could offset or address these barriers and further improve health outcomes for enrolled families and young children. Families’ social contexts will be critical to the design and delivery of such interventions, given the key role they play in the provision (or not) of support and resources. Postpartum mothers may also be experiencing rapid changes to their social connections, at a time when social support is much needed [9–11]. To date, research on the interplay of social contexts and dietary intake among recipients of HVP services has been limited [11–13].

This broader social context includes one’s social network, comprised of family members, friends, colleagues, and other significant social ties, that wields considerable influence over health behaviors, including dietary patterns [14, 15]. In line with the social buffering hypothesis [16], the provision of health-related social support (i.e., “health support”) has been shown to buffer against barriers and promote access and engagement in health-promoting practices [17–20]. Social network members may offer support in encouraging engagement in health behaviors, or provide health advice and information [17–19, 21, 22]. However, social network members may also undermine health or increase barriers to healthy dietary behaviors [23–26]. These “health underminers” may operate deliberately, or unintentionally through mechanisms like negative social influence [23–26]. Both health supporters and health underminers influence diet outcomes, and may be even more impactful when they are key network members, such as a household member or a romantic partner [20, 27].

Given the role of social networks in supporting or undermining health practices, it is valuable to understand what gives rise to these social phenomena among mothers enrolled in HVPs. Extensive research indicates that general social support arises from a complex set of interactive features of social network structures and social norms [26, 28–30]. Regarding health support specifically, a few studies found the following social features were predictors of support provision or receipt: the age and health status of the recipient of health support; the gender, emotional closeness, social role, and physical proximity of the provider of health support; and the size and health norms of the recipient’s overall social network [28, 30, 31]. We identified no studies seeking to examine which social features give rise to health undermining in social networks.

Mothers enrolled in HVPs are a priority population, and the postpartum period in particular is an opportune window to motivate and enable healthy changes in dietary behavior [32]. Further, the postpartum period is a time of rapidly changing social dynamics, such as the strengthening of family relationships, which have been linked to mothers’ health outcomes [11–13]. HVPs provide support and resources that can facilitate social support and foster a healthy diet, but research is needed on the social contexts in which these services are provided. Herein, this study explores the social networks of mothers served by a HVP in Southern California, and how characteristics of mothers and their social networks are associated with (Aim 1a) overall health support and health undermining within their network, and (Aim 1b) the likelihood of a given network member providing health support and health undermining (e.g., more females in one’s network may be linked with a greater number of overall supporters, but does *being* female make someone more likely to be a supporter?). Additionally, this study tests (Aim 2) how health support and health undermining, and features of supporters and underminers, are associated with mothers’ dietary intake.

## Methods

This study was embedded within a two-parallel arm randomized controlled trial, called “Healthy Habits” (R01HD092483). The details of the parent trial have been described elsewhere [32], but generally evaluated the impact of incorporating new healthy eating and activity intervention material into existing HVP curricula. Healthy Habits was delivered at two community-based organization sites, Antelope Valley Partners for Health (AVPH) and Children’s Bureau (CB), who are both established providers of the Healthy Families America HVP. Eligible mothers and infants are provided services for up to the first 2 years after the infant’s birth. AVPH and CB serve racially and ethnically diverse families living in Antelope Valley California, which is an area in the northern high-desert region of Los Angeles County with a population of around 434,000 individuals. Both sites initiate HVP services soon after birth and mothers must meet a number of risk criteria (e.g., low-income, housing instability) to receive services. Most clients are referred by a healthcare provider, a community partner, or recruited at bedside or through outreach events. All referrals are assessed and screened by family resource specialists or supervisors for programmatic eligibility.

The Healthy Families America curriculum is delivered by home visitors in the home on a weekly or monthly basis, depending on how long a client has been enrolled. The curriculum focuses on strengthening parent–child relationships and family functioning, promoting positive child development, and linkage to community resources (e.g., medical providers, financial/housing assistance, childcare, substance abuse treatment, community programs, and government benefits including WIC). Healthy Families America accredited home visitors are matched to families on cultural background and language (English or Spanish), to provide culturally sensitive services. Home visitors receive rigorous training in cultural competency, substance abuse, reporting child abuse, domestic violence, linkage to services in their community, and more.

The present study focuses on data collected at the baseline of the Healthy Habits trial, among mothers who were enrolled in the above-mentioned HVPs but who had not yet received any study intervention material. All study procedures were approved by the Institutional Review Board of University of University of Southern California (HS-18–00025).

### Participants

The Healthy Habits study enrolled mothers and children who were HVP clients at our partner sites on a rolling basis from November of 2018 to May of 2022. Most HVP clients at this partner site were enrolled immediately after the child’s birth. To be eligible for the Healthy Habits study, the family needed to have been receiving HVP services for at least 2 months, and the “index child” enrolled in the HVP needed to be less than 24 months old. Thus, at the time of enrollment in the Healthy Habits study, and baseline data collection, study participants had been receiving HVP services from time periods ranging from 2 months to 2 years, while the average time was 9.6 months (SD = 7.0 months).

The present study uses cross-sectional, baseline data collected prior to the delivery of the Healthy Habits intervention. Thus, mothers had been receiving HVP services prior to data collection, but had not been receiving the Healthy Habits intervention. The baseline sample included 76 mother–child dyads, and this analysis focuses on the mothers’ data only. The baseline sample size was smaller than planned because the COVID-19 pandemic halted and slowed the enrollment of mothers into HVP for 14 months. However, a small sample size is not uncommon in studies utilizing social network data, because in-depth data is time intensive to collect, and social network ties are also units of analysis [33]. Additionally, 7 mothers were excluded from the analysis due to missing or incomplete social network data.

### Protocol

Eligible clients were approached by their home visitor, and if interested they signed a release of information form that was then given to the study team. A trained study team member contacted the client to give more information on the study and obtain informed consent of the mother, on behalf of herself and her infant. Once enrolled, trained assessment workers administered the self-report questions either via Zoom or in person, and verbally in English or Spanish, depending upon the preferred language. Participants were compensated $30 for this baseline assessment.

### Measures

The specific questions and scales used for the measures are outlined in detail in Supplemental Table I.

#### Individual‑Level Covariates and Predictors

Although a variety of complex multilevel factors influence dietary intake among low-income mothers [4–8], the focus of this study is on social networks. Given our small and somewhat homogeneous sample, we tested key individual-level covariates and predictors including demographics, food insecurity, and breastfeeding status, which are strongly linked with maternal diet [34].

Participants’ ethnicity, race, maternal age, infant age, education, and annual household income were assessed using a brief demographic questionnaire. *Food insecurity* was measured using the six-item USDA Household Food Security Survey [35], that assesses experiences of food insecurity (e.g., skipping meals due to concerns over insufficient money or food) over the past 12 months. Respondents were categorized as having: (1) food security; (2) low food security; and (3) very low food security. *Breastfeeding status* was measured by asking how many months the participant breastfed the “index child” in the HVP and subtracting this number from the participating child’s age in months to determine if the mother had breastfed this child within the past month (yes/no).

#### Dietary Intake

Participants self-reported their intake frequency for fruits and vegetables using valid measures adapted from the National Health and Nutrition Examination Survey (NHANES) [36]. Sugar-sweetened beverage and juice (SSB) intake was captured using a 4-item scale adapted from Lundeen et al. [37]. As these food frequency questionnaires do not collect fine-grained dietary data, categorization is more appropriate than trying to estimate the number of servings [36]. Thus, based on the distribution of answers, intake for fruits was categorized as less than one serving per day vs. 1 or more servings per day, as was intake of vegetables; while intake of SSBs was categorized as 0 servings per day vs. more than 0 servings per day.

#### Social Network

Mothers’ social networks were assessed using personal or “egocentric” social network methods that capture subjective information from mothers about the social connections they have, and the characteristics of these network members [38]. First, a name generator was used in which participants were asked to identify 15 adults whom they considered to be the most important people in their lives during the past 6 months, and with whom they had regular contact [38, 39]. Subsequently, participants reported on the characteristics of each network member, including if they were sources of health support or health undermining, their demographics, and other key attributes. Characteristics of network members, or of the network as a whole, were assessed if they had been found to predict health support or undermining in previous quantitative or qualitative studies [20, 28, 30, 31]. For example, previous quantitative studies identified network members’ gender, emotional closeness, social role, physical proximity, as well the overall network gender composition and emotional closeness as attributes predicting health support [28, 30, 31]. These studies have also shown the importance of concordant healthy behaviors and attributes in determining health support, so network members’ weight status was also explored [31]. Further, qualitative studies have suggested the importance of some of these features in association with health undermining [20].

##### Health Supporters and Underminers

Network members who provided health support were identified through the question: *In the past 6 months, who helped you or encouraged you to have a healthy lifestyle, to eat healthy foods, or to be active?* Network members who engaged in health undermining were identified through the question: *In the past 6 months, who made it difficult for you to have a healthy lifestyle, eat healthy foods, or be active?* If the participants indicated that the network member made it difficult, they were asked the open-ended question of *Why?* Each network member was coded as being a health supporter (1) or not (0), and as a health underminer (1) or not (0). The total *number of health supporters in their network* (or number of health underminers) was computed by summing the total number of health supporters (or health underminers) among their network members (15).

##### Gender

Participants reported the *gender of each network member* (female, male, unsure/prefer not to answer). The *proportion of females in their network* was computed by dividing the total number of females by the number of network members (15).

##### Emotional Closeness

Participants reported their *emotional closeness to each network member* on a 4-point Likert scale, with higher scores indicating higher emotional closeness. The *average emotional closeness of their network* [31] was computed as the mean score for the emotional closeness of all network members (e.g., higher scores indicating more emotionally closed networks).

##### Social Role

Participants reported on each network member’s relationship to themselves (e.g., the mother, father, sister, and friend). The *social role of each network member* was recategorized as family (e.g., both biological and non-biological kin, such as husband, romantic partner, parents, aunts, uncles, and mothers-in-law), friends, other personal relationships (i.e., neighbor, roommate, coworker), and resource-based relationships (e.g., home visitors, educator, therapist, pastor, and sponsor).

##### Perception of Overweight or Obesity

The *perceived weight status of each network member* was measured using a validated figure silhouette scale [40]. Each network member was designated as (0) perceived as being underweight or normal weight or (1) perceived as having overweight or obesity. The *proportion of people perceived as having overweight or obesity in their network* was computed by dividing the total number of network members who were perceived as having overweight or obesity by the total number of network members (15).

##### Physical Proximity

Participants reported on where each network member lived, in relation to them, and responses were used to identify if each network member lived in the *same household* (yes/no), or in the *same household or neighborhood* (yes/no).

##### Characteristics of Health Supporters and Health Underminers

As others have shown that certain types of health supporters are most influential [28, 30, 31], we also computed and tested the social role of the supporter/underminer (e.g., family member who is a supporter, partner who is a supporter) and the physical proximity of the supporter/underminer (e.g., household member is a supporter).

##### Network Structure

For each pair of network members that had been listed, participants indicated whether they knew each other. This was used to compute density and transitivity, two social network structure features that have been linked extensively to general social support but not yet to health support or undermining [28, 33, 41, 42]. *Density* describes the proportion (0–100%) of participant’s network members that are connected to one another (i.e., the number of pairs who knew each other), relative to the total number of potential connections (i.e., the total number of pairs). *Transitivity* represents the phenomenon of “a friend of a friend is my friend.” Transitivity is computed by examining the presence or absence of ties between triads of network members within the respondent’s social network. A triad is considered transitive when all three members are connected to each other (e.g., A knows B, and B knows C, and A knows C). Transitivity was scaled to represent a 10% increase. A higher network density represents a network that is overall very densely connected (i.e., most individuals in the network know all the other network members), while transitivity represents a network that has subgroups that are densely connected (i.e., the network can be divided into subgroups in which all network members know each other, but they don’t necessarily know individuals in the other subgroups). Both higher density and transitivity have been linked with greater social support [28, 33, 41, 42].

### Analytic Methods

Social network variables were computed using R. Because personal social network studies focus on the social networks of individuals who are in a study sample, participants are assumed to be independent, and standard statistical methods that assume data independence, like correlations and regressions, are employed. All statistical tests were performed using SAS v9.4. Means and standard deviations were computed for all continuous predictors and continuous outcomes, and frequencies and percentages were computed for all categorical predictors and outcomes (Tables 1 and 2).

Pairwise tests (e.g., Pearson’s R) were used to initially explore potential relationships. For aim 1a, linear regression models were used to predict the total number of health supporters in one’s network, and logistic regression models were used to predict whether participants had 0 vs. 1 or more health underminers (since most participants had zero or one health underminers). For aim 1b, network member characteristics were nested within mothers, and separate multilevel logistic regression models predicted: (1) odds of a network member being a health supporter; and (2) odds of a network member being a health underminer. For aim 2, separate logistic regression models predicted odds of: (1) daily fruit consumption (1 or more serving per day vs. less than one per day); (2) daily vegetable consumption (1 or more servings per day vs. less than one per day); and (3) daily SSB consumption (more than zero servings per day vs. no servings per day).

Ethnicity (Hispanic vs. Non-Hispanic), maternal age, and education level (high school, GED or less vs. some technical training, college or more) were included as control variables in the analyses. For all three aims, a stepwise approach was used to specify the regression models. Predictors were retained in the final model when they were statistically significant (*p* < 0.05) and significantly improved the fit of the model (e.g., the log likelihood, AIC, or BIC).

## Results

### Descriptive Statistics

Descriptive statistics for mother characteristics (*n* = 69) are summarized in Table 1. 72.5% of mothers identified as Hispanic or Latina. Nearly one-half of mothers (49.3%) consumed one or more servings of fruits per day, 46.4% consumed one or more servings of vegetables per day, and 63.8% consumed at least one SSB each day. Just over half the mothers (55.0%) had a household income below $30,000, and 59.4% were either married or living with a romantic partner (all romantic partners were male). A little less than half of mothers (49.3%) had some college or a college degree.

Mother’s social network characteristics are summarized in Tables 1 and 2. As every mother listed 15 network members, there were a total of 1035 network members, with 227 (21.9%) identified as health supporters and 56 (5.4%) identified as health underminers. Most mothers (95.7%) had at least one health supporter in their social network, while 55.1% had at least one health underminer. On average, mothers’ social networks had a density of 0.70 (SD = 0.2), meaning that 70% of network member pairs knew each other; indicative of densely connected networks. The average network transitivity was 0.8 (SD = 0.2), indicating a high level of network cohesion. The average emotional closeness score was 1.7 (SD = 0.1) and on average, 50% of network members were perceived by the mother as having overweight or obesity.

In addition, participants were asked to describe how network members undermined their health practices. Among the 46 responses given, four themes emerged to characterize the type of undermining behaviors: (1) the network member ate or cooked unhealthy foods (e.g., eats junk foods, fries a lot of foods) (*n* = 24); (2) the network member provided unhealthy foods or invited the participant to eat unhealthy foods (e.g., brings too many drinks with sugar, takes me out to eat) (*n* = 13); (3) more broad statements about the network member being an unhealthy influence on eating (e.g., bad influence on eating habits) (*n* = 3); and (4) other/unclear (e.g., personal issues, fried foods) (*n* = 6).

### Regression Results for Aim 1a: Predictors of Overall Network Health Support and Health Undermining

The results of the final regression model for Aim 1 are summarized in Table 3. For health support, mothers with household incomes ≥ $30,000 had a higher number of health supporters in their network than mothers who had a household income of < $10,000 per year (*b* = 1.36, *p* = 0.03). No other individual or network characteristics predicted health support.

For health undermining, none of the mothers’ characteristics were associated with having one or more health underminers in their social network. At the network level, having a higher proportion of females in one’s network was associated with lower odds for having one or more health underminers (OR = 0.01, *p* = 0.03).

### Regression Results for Aim 1b: Predictors of Network Members’ Provision of Health Support and Health Undermining

The results of the final regression model for Aim 1b are summarized in Table 4. For health support, any given network member was significantly more likely to be a health supporter if the mother had a household income of $10,000–29,999 (OR = 2.9, *p* < 0.01), or a household income of ≥ $30,000 (OR = 2.78, *p* < 0.01), compared to mothers with household incomes of < $10,000. Network members were significantly more likely to be health supporters if they were romantic partners (OR = 3.41, *p* < 0.001), resource-based connections (OR = 3.46, *p* < 0.01), or if they lived in the same household or neighborhood (OR = 1.68, *p* < 0.05). Network members were significantly less likely to be health supporters if they were perceived as having overweight or obesity (OR = 0.52, *p* < 0.001). Finally, mothers’ social network transitivity positively predicted the likelihood of a network member being a health supporter: in social networks that were 10% more transitive, any given network member was more likely to be a health supporter (OR = 1.25, *p* < 0.05).

For health undermining, mothers’ characteristics did not predict whether a given network member was likely to be a health underminer. At the social network level, network members were significantly more likely to be underminers if they were romantic partners (OR = 8.93, *p* < 0.0001), or if they were perceived by the mother as having overweight or obesity (OR = 3.98, *p* < 0.001).

### Regression Results for Aim 2: Association Between Health Support and Health Undermining with Mothers’ Dietary Intake

Results for the final regression models for Aim 2 are summarized in Table 5. We found that mothers who had at least one health supporter who was *living in their household or neighborhood* were more likely to consume one or more servings of vegetables per day (OR = 3.0, *p* < 0.05), and less likely to consume any daily servings of SSBs (OR = 0.29, *p* < 0.05). Mothers who had at least one health supporter *who was a family member* were more likely to consume one or more servings of fruit per day (OR = 3.2, *p* < 0.05). Health-undermining characteristics were not significantly associated with dietary intake.

## Discussion

This study examined the personal social networks and dietary intake of racially and ethnically diverse mothers enrolled in a HVP in Los Angeles, CA. First, we found that mothers’ intake of fruits and vegetables was similar to national levels [43], despite their intersection of potential risk factors for poor nutrition. This could be because most of the mothers were currently or recently postpartum, a time when mothers are more likely to improve nutrition for the sake of breastfeeding and postpartum health [34]. This could also be due to the support and resource referrals they receive through HVPs. With regard to the primary focus of this study, we found that several maternal and social network characteristics were linked with having health supporters and health underminers (Aims 1a and 1b). Having certain types of health supporters was associated with some healthier dietary patterns, but we found no association between health underminers and mothers’ dietary intake (Aim 2).

Almost all the mothers in this study named at least one health supporter in their personal social network, and nearly half had 3 or more supporters, that were mostly family members or friends, and female. This finding is encouraging, and for the newer mothers, it could have been due in part to the postpartum period, a time when women are more likely to try to seek social support from their networks [11, 44]. However, mothers with the lowest number of health supporters were those with the lowest incomes. This is consistent with other literature and demonstrates that individuals who need the most social support are often the ones who receive the least [29, 45].

Another positive finding was that mothers had fewer health underminers than health supporters, and no mothers named more than two health underminers. Interestingly, mothers with more females in their personal social network were less likely to have a health underminer in their network. There is extensive literature documenting that females are more likely to both seek and provide social support and that they are invested in the health of their social networks [31, 46, 47]. Thus, networks with more females may have stronger pro-social norms that are less accepting of undermining. Future work should explore if the concern women show for their social networks’ health and well-being improves network-level social norms related to health undermining, discouragement, and conflict.

There were also characteristics associated with network members’ likelihood of providing health support. Network members were more likely to be health supporters if they lived in the same household or neighborhood, highlighting the importance of physical proximity in facilitating social support provision, as has been found in other studies [10, 28]. Additionally, any given network member was more likely to provide health support if they were embedded in a more transitive network—meaning their network was more socially cohesive (i.e., where network members who had mutual ties were more likely to be connected, creating an overall more well-connected network) [28, 33]. This is aligned with the literature on social cohesion and general social support, which has found that cohesive, transitive networks tend to have stronger social norms and rules about the exchange of support among members [28].

We also found that resource-based ties, such as home visitors, therapists, and other health care professionals who provide health support as part of their profession, were identified as health supporters. As mothers may receive more regular and ongoing health care during the peri and newly postpartum period, this finding is not surprising. However, it is notable that these individuals were identified by these mothers as important members of their social networks in the past 6 months. This indicates that these professionals have a window of time to occupy important “brokering” positions, where they may serve as a bridge that connects mothers with outside individuals, resources, and information, that may then be shared back to mothers’ social networks [48]. By doing so, these brokers are providing information and resources to communities that have traditionally lacked access to health services [48]. These brokers may be leveraged in interventions that aim to improve health outcomes in marginalized communities [48], as is being done in the intervention associated with this study [32]. Notably, naming one of these professionals as a health supporter was not associated with healthier dietary patterns among mothers. Many of these health care professionals presumably already address the importance of diet in ongoing conversations around breast-feeding and postpartum health. As such, this suggests that these professionals may benefit from further resources and training, such as evidence-based nutrition curricula they can share with clients. As the HVP curriculum may vary between program models, this additional resource and training may be especially beneficial for home visitors in models that tend to focus more on healthy child-feeding practices and less on other aspects of maternal nutrition.

When mothers perceived a network member as having overweight or obesity, they were less likely to identify them as a source of health support and more likely to identify them as a source of health undermining. As obesity is linked with a myriad of barriers over the life course such as low income or food insecurity [49, 50], these network members with obesity may be constrained by their own socio-ecological barriers that inhibit their capacity to provide support.

Finally, romantic partners were more likely to be both health supporters and health underminers. Romantic partners tend to become more similar in health behaviors and health status over time, due to many processes, that include mechanisms of social influence [47, 51]. For individuals trying to improve their lifestyle, having a supportive romantic partner who can be a positive social influence is often a key factor to success, though unwanted or coercive support is detrimental [47, 51]. The right kind of health support from partners may be vital for mothers in HVPs, as work with this group and others have emphasized romantic partners as particularly influential social ties, with the capacity for both encouraging and deterring improvements in health behaviors [20, 52, 53]. As HVPs regularly engage other parents, romantic partners, and family members in their services, there may be opportunities for them to provide additional guidance on how to manage and reduce health undermining from these partners.

Unexpectedly, the overall number of health supporters and underminers in mothers’ social networks was generally not associated with their dietary intake, though health support from certain types of network members was beneficial. These findings with regard to health undermining may be driven by the fact that all mothers had at least one health supporter, and the presence of support may have counteracted the negative effects of health undermining, as has been found in other studies [54]. The lack of a relationship between overall health support with diet outcomes could be due to the complexity of social support mechanisms among women with low income [55, 56]. These women who have constrained resources are likely to be mostly connected to other individuals with limited resources, because social networks are known to have strong “homophily” where connections are more likely among people with similar sociodemographic characteristics [56, 57]. Their network members may not have adequate means for providing support, thus they may provide support in ways that are not needed or are not beneficial to health, creating detrimental “mismatches” in support [58, 59].

Though overall network health support was not associated with dietary intake, health support from certain types of network members was associated with better dietary outcomes. In line with findings of previous studies [10, 27], having a supporter who lived in one’s household or neighborhood, or who was a family member, was linked with better dietary patterns. This emphasizes the importance of closeness, both in terms of physical and relational proximity, in determining the availability of support, information, and resources from one’s social ties [10, 27, 32, 60, 61], and the importance and impact of this support on health behaviors. As such, interventions that holistically target family and community systems may be most effective at promoting healthy dietary and weight outcomes.

### Strengths and Limitations

Most studies focus on general perceptions of support, rather than integrating more comprehensive measures of network support and considering broad social networks. This study’s most notable strength is the use of rich and in-depth social network data for a broad set of network members (i.e., 15 individuals). This study is among the first to examine the network characteristics associated with health support and health undermining, and the link between both health support and health undermining on dietary intake. Furthermore, this study makes use of a unique, ethnically diverse sample that has been underrepresented in research and has an increased risk of poor dietary patterns and diet-related diseases.

Aim 2 of this study is limited by a small sample size, due in part to the interruption of enrollment during the COVID-19 pandemic. However, our power analyses still indicated sufficient power to detect medium-size effects, and a small sample size is not uncommon in research on social networks. As this sample of mothers is from one HVP site, the findings of this study may not be generalizable to mothers in all HVPs across the U.S. Additionally, these data are based on self-reported data, which is subject to self-report bias. As social network data was self-reported, these findings have implications for the *perceptions* of health support and health undermining within networks, rather than actualized support. Finally, this study utilizes food frequency questionnaires, which have some notable limitations such as being subject to self-report bias and only being able to approximate dietary intake [62], despite being one of the more predominantly used tools to assess patterns of intake for key food types. To address the limitations of this study and expand on these findings, future studies should aim to explore the longitudinal relationships between social networks, health support, and dietary patterns in mothers enrolled in HVPs nationally.

### Implications

Our findings, along with an extensive body of social network studies, highlight the key influence of important others on dietary intake [32, 48, 63]. While social context matters for the uptake and sustainability of health interventions, it is often overlooked in implementation. Integrating social network intervention strategies into HVPs may help program recipients manage social networks that are a mix of health supporters and health underminers, and navigate the multiplicity of social ties who were simultaneously sources of both support and undermining. For example, HVPs could integrate new intervention components like those described in the Healthy Habits model [32], which includes both cognitive-behavioral and social network-based approaches to improve nutrition. Specifically, Healthy Habits incorporates cognitive-behavioral techniques centered on habit formation, such as helping parents modify dietary cues in their home environment, while also using social-network-based techniques like engaging whole households and families in curriculum and activities. Further, Healthy Habits also explicitly builds adjacent community-based social networks to support health through communal classes where caregivers can learn, exchange, and receive support from other caregivers. As the postpartum period is a time that facilitates rapid social change [11–13], this is a key opportunity for clients to develop new social ties who share similar health-based goals, leading to the formation of new health supporters [32]. Lastly, in addition to the Healthy Habits model, HVPs have the potential to build upon their current curricula that help clients build healthy relational and communication skills, and provide curricula that specifically target skills around health-related conflict resolution. In summary, HVPs offer a unique delivery infrastructure for intervention models that include both cognitive-behavioral and social network-based approaches to improve nutrition and other health outcomes.

## Supplementary Material

Supplementary material

The online version contains supplementary material available at https://doi.org/10.1007/s40615-025-02286-z.

## Figures and Tables

**Table 1:** Descriptive statistics for mothers enrolled in a HVP in Los Angeles County (*N* = 69)

	Frequency	Percent
Income		
Under $9999 per year	21	30.4
$10,000–29,999 per year	17	24.6
$30,000 or above	20	29.0
Unsure/declined to answer	11	15.9
Age		
16–25 yrs old	16	23.2
26–35 yrs old	42	60.9
36 + yrs old	11	15.9
Infant age		
Under 6 months	28	36.8
6 to 11 months	21	27.6
12 months and older	27	35.5
Recently breastfeeding		
Yes	33	43.4
No	43	56.6
Education		
High school/GED or less	35	50.7
Some colleges or more	34	49.3
Ethnicity		
Hispanic, any race	50	72.5
Non-Hispanic/decline/unsure	19	27.5
Food insecurity		
Food secure	47	61.8
Low food security	18	23.7
Very low food security	11	14.5
Dietary intake		
Fruit intake (1 + servings per day)	34	49.3
Vegetable intake (1 + servings per day)	32	46.4
SSB intake (> 0 servings per day)	44	63.8
Social network characteristics		
1 + Health supporter	66	95.7
1–2 supporters	33	47.8
3–4 supporters	19	27.5
4–7 supporters	14	20.3
1 + Health underminer	38	55.1
1 Underminer	20	29.0
2 Underminers	11	15.9
	Mean	Std dev
Number of health supporters	2.9	2.0
Number of health underminers	0.6	0.7
Density	63.0	23.6
Transitivity	77.7	15.4
Average emotional closeness	1.7	0.4
Proportion of females	0.7	0.1
Proportion overweight or obese	0.5	0.2

**Table 2:** Characteristics of mothers’ social network members, health supporters, and health underminers

Social network member characteristic	All network members (*n* = 1035) *N* (%)	Supporters (*n* = 227) *N* (%)	Underminers (*n* = 56) *N* (%)
Female	697 (67.4)	162 (71.4)	33 (58.9)
Social role			
Family	553 (53.7)	112 (49.3)	24 (43.6)
Partner	51 (5.0)	24 (10.6)	14 (25.5)
Friend	345 (33.5)	70 (30.8)	15 (27.3)
Resource	37 (3.6)	13 (5.7)	–
Other	44 (4.3)	8 (3.5)	2 (3.6)
Physical proximity			
Household member	100 (9.7)	35 (15.4)	16 (28.6)
Same household or neighborhood	270 (26.1)	81 (35.7)	27 (48.2)
Has overweight or obesity	547 (53.0)	94 (41.4)	44 (78.6)

**Table 3:** Aim 1a: results of regression models to identify predictors of overall network health support and undermining

	Support			Undermining	
	b	SE	*p*-value	OR (95% CI)	*p*-value
Intercept	1.70	1.17	0.15	4.90	0.3
**Mother characteristics**					
Hispanic	0.42	0.56	0.46	1.86 (0.53, 6.59)	0.2
Age	0.03	0.04	0.43	1.01 (0.94, 1.11)	0.3
Education (ref: Some college or more)					
High school/GED	0.13	0.50	0.80	1.18 (0.38, 3.69)	0.9
Household income (ref: under $10,000 per year)					
$10,000–29,999	1.13	0.66	0.09	NS	–
$30,000 or above	1.36	0.64	0.03	NS	–
Unsure/declined **Social network characteristics**	1.13	0.74	0.13	NS	–
Proportion of females	NS	–	–	0.010 (< 0.001, 0.70)	0.03

**Table 4:** Aim 1b: results of multilevel regression models to identify predictors of network members’ provision of health support and health undermining

	Supporter		Underminer	
	OR (95% CI)	*p*-value	OR (95% CI)	*p*-value
Intercept	1.04	0.01	0.002	< 0.0001
**Mother characteristics**				
Hispanic (yes)	0.70 (0.33, 1.48)	0.35	1.72 (0.62, 4.76)	0.30
Mother age	1.01 (0.97, 1.06)	0.56	1.05 (0.98, 1.11)	0.16
Education (ref: some college or more)				
High school education/GED	1.17 (0.61, 2.23)	0.64	1.18 (0.52, 2.69)	0.69
Income (ref: less than $10,000 per year)				
$10,000–29,999	2.90 (1.25, 6.71)	0.01	NS	–
$30,000 or above	2.78 (1.23, 6.26)	0.01	NS	–
Unsure/declined	2.39 (0.93, 6.12)	0.08	NS	–
**Social network characteristics**				
*Network member characteristics*				
Relation (ref = friend)				
Family	0.86 (0.58, 1.28)	0.46	0.90 (0.44, 1.84)	0.77
Partner	3.41 (1.68, 6.90)	0.00	8.93 (3.68, 21.65)	< 0.0001
Resource	3.46 (1.49, 8.01)	0.00	NS	–
Other	0.92 (0.35, 2.44)	0.86	0.63 (0.13, 2.99)	0.56
Same household or neighborhood	1.68 (1.10, 2.56)	0.02	NS	–
Overweight or obese	0.52 (0.37, 0.73)	0.00	3.98 (1.93, 8.20)	0.00
*Overall network characteristics*				
Transitivity	1.25 (1.04, 1.5)	0.04	NS	–

**Table 5:** Aim 2: results of regression models to identify association of network health support and health undermining with mothers’ dietary intake

	1 + Veg per day (Yes)		1 + Fruit per day (Yes)		> 0 SSB per day (Yes)	
	OR (95% CI)	*p*-value	OR (95% CI)	*p*-value	OR (95% CI)	*p*-value
Hispanic	0.33 (0.95, 1.14)	0.08	3.19 (0.88, 11.5)	0.08	0.81 (0.22, 2.95)	0.76
Age	0.99 (0.92, 1.07)	0.85	1.00 (092, 1.08)	0.91	1.01 (0.93, 1.08)	0.89
Education (ref: Some college or more)						
High school/GED	0.60 (0.21, 1.73)	0.35	1.14 (0.39, 3.35)	0.81	0.55 (0.18, 1.64)	0.28
**Social network predictors**						
Neighborhood supporter^a^ (yes)	3.00 (1.03, 8.77)	0.04	NS	–	0.29 (0.09, 0.89)	0.03
Family supporter^b^ (yes)	NS	–	3.15 (1.0, 9.94)	0.05	NS	–

a Neighborhood supporter: network member who was identified as both a provider of health support and who lived in the same household or neighborhood

b Family supporter: network member who was identified as both a provider of health support and who was a family member
