# Supplementary material for: Social Networks, Health Support, and Dietary Intake in Mothers Receiving Home Visiting Services
Source: J Racial Ethn Health Disparities. 2025 Jan 22;13(2):737–48. doi: 10.1007/s40615-025-02286-z (PMC12863036; doi:10.1007/s40615-025-02286-z)
Supplement: Supplementary file 1 — Supplementary file1 (DOCX 17 KB) [file 40615_2025_2286_MOESM1_ESM.docx]

| **Table SI. Questions and Scales used for Measures** | | |
| --- | --- | --- |
| **Variable** | **Scale or questionnaire** | **Response Options** |
| **Demographics and Individual-Level Predictors** | | |
| Race, ethnicity, maternal age, infant age, education and annual household income | Standard demographic questionnaire |  |
| Breastfeeding status | How many months did you breastfeed (infant name)? | Open ended |
| Food insecurity | USDA Household Food Security Survey |  |
| **Dietary intake** | | |
| Fruit intake | “*During the past month, how often did you eat fruits with your meals or as a snack?”* | 1) None; (2) 1 to 3 times a month; (3) 1 to 3 times a week; (4) 4 to 6 times a week; (5) 1 time a day; (6) 2 times a day; (7) 3 or more times a day; or (8) DK/Refused. |
| Vegetable intake | *During the past month, how often did you eat vegetables with your meals or as a snack?”.* | 1) None; (2) 1 to 3 times a month; (3) 1 to 3 times a week; (4) 4 to 6 times a week; (5) 1 time a day; (6) 2 times a day; (7) 3 or more times a day; or (8) DK/Refused. |
| SSB Intake | 4-item scale adapted from Lundeen et al. [37] assessing consumption of (1) soda; (2) coffee and tea; (3) sports and energy drinks; and (4) fruit drinks or juices | (1) None; (2) 1 to 3 times a month; (3) 1 to 3 times a week; (4) 4 to 6 times a week; (5) 1 time a day; (6) 2 times a day; (7) 3 or more times a day; or (8) DK/Refused. |
| **Social Network Measures** | | |
| Name generator | *I’d like you to name 15 adults that you know and who know you, and who you consider to be the most important people in your life in the past 6 months.* | Open ended response |
| Health support | *“In the past 6 months, who helped you or encouraged you to have a healthy lifestyle, to eat healthy foods or to be active?”.* | List of network members |
| Health undermining | “*In the past 6 months, who made it difficult for you to have a healthy lifestyle, eat healthy foods, or be active?”.* | List of network members |
| Gender | *What is Person X’s gender?* | Male; Female; Other |
| Emotional closeness | *How much do you agree or disagree that you and Person X are close to each other?”* | (0) Strong disagree; (1) Disagree; (2) Agree; (3) Strongly Agree |
| Social role | *What is Person X’s relationship to you?* | Spouse; brother; sister; mother; father; cousin; aunt; uncle; friend; neighbor; other (please specify) |
| Perception of overweight/obesity | “*Which of these images best reflects Person X’s current appearance*?” | Four silhouettes were presented as the response options, representing underweight, normal weight, overweight, and obese figures. |
| Physical proximity | *“Compared to YOU, where does Person X live?”.* | Same household; same neighborhood; same state; same country; another country |
| Network structure | Does person i and person j know one another?” | Yes; no; don’t know |
